# The Interazygos Vein: A Case Report and Review of Preaortic Hemiazygos Anastomoses

**DOI:** 10.7759/cureus.83800

**Published:** 2025-05-09

**Authors:** Aarti N Sahai, Liliana M D'Alesio, Andrew L Dugaesescu, Jay M Bauman

**Affiliations:** 1 Anatomical Sciences, Saint Louis University School of Medicine, St. Louis, USA

**Keywords:** anastomosis, anatomical variation, azygos, hemiazygos, interazygos

## Abstract

During routine dissection of the thoracic cavity, a rare variation of the azygos system of veins was observed. The hemiazygos vein and the accessory hemiazygos vein generally course posterior to the aorta to form anastomoses with the azygos vein. However, in this case, the hemiazygos vein and the accessory hemiazygos vein anastomosed together into a vessel that crossed anterior to the thoracic aorta before draining into the azygos vein on the right side of the thoracic cavity. This variation is also known as an "interazygos vein."

While the venous system is recognized for its variability, a preaortic anastomosis is particularly rare. A literature review was conducted to investigate the morphology of previous reports of preaortic hemiazygos anastomoses and interazygos veins. It was determined that the term "interazygos vein" encompasses a range of morphological variations. Awareness of the interazygos vein is important clinically to avoid mediastinal misdiagnoses and surgical complications.

## Introduction

The azygos system of veins handles the drainage of the posterior thoracic wall. The azygos vein ascends the right side of the vertebral column, receives blood from the intercostal veins emerging from the right intercostal spaces numbered 5-11 directly, then drains into the superior vena cava. The hemiazygos vein ascends on the left side, drains the left posterior intercostal spaces 9-11, then crosses to the right at the level of the ninth thoracic vertebra, posterior to the aorta, to empty into the azygos vein. The accessory hemiazygos vein receives venous blood flow from the left posterior intercostal spaces 4-8, then crosses posterior to the aorta at the level of the eighth thoracic vertebra to drain into the azygos vein as well [1].

The azygos system is characterized by its high likelihood of possessing anatomic variation in general, and some specific variations are particularly common. In 40% of cases, the accessory hemiazygos vein opens directly into the hemiazygos vein rather than crossing over to the azygos vein. The accessory hemiazygos vein has also been observed to communicate with the left superior intercostal vein in 57% of cases [2]. Systems have been proposed to classify the range of variations encountered in the azygos veins [3]. However, these classifications are all premised on a "retroaortic" (i.e., posterior to the aorta) course of the hemiazygos and accessory hemiazygos veins.

In rare cases, an anastomosis of the hemiazygos and accessory hemiazygos veins passes anterior to the aorta ("preaortic") to drain into the azygos vein [4]. This variation has become known as an "interazygos vein" [5,6]. In addition to contributing a report, this paper reviews the morphology of all cases cited as either a preaortic hemiazygos anastomosis or an interazygos vein. Recognition of the interazygos vein is particularly relevant to radiologists and surgeons due to its potential to mimic pathologies of the mediastinum [7].

## Case presentation

A rare anatomic variation was discovered during routine dissection in a medical gross anatomy course. The whole body donor was an 88-year-old male who died of respiratory failure, intraparenchymal hemorrhage, and cerebral venous thrombosis. All donor bodies were obtained through the Saint Louis University Gift Body Program of the Center for Anatomical Science and Education (CASE) with signed informed consent from the donors. The CASE Gift Body Program abides by all rules set forth by the Uniform Anatomical Gift Act.

**Figure 1:**
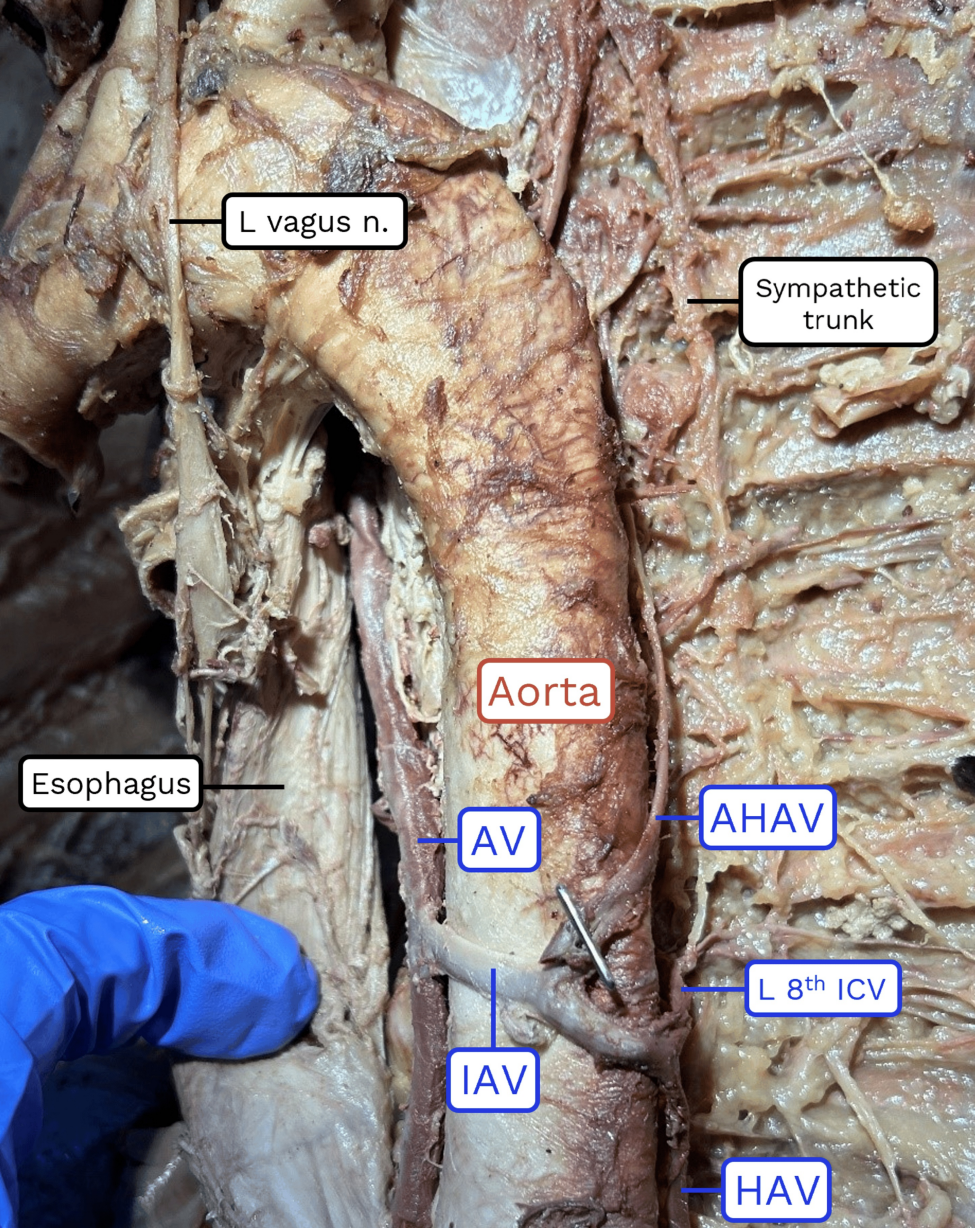
Photograph of the observed anatomic variation. AV: azygos vein; IAV: interazygos vein; HAV: hemiazygos vein; AHAV: accessory hemiazygos vein; ICV: intercostal vein.

**Figure 2:**
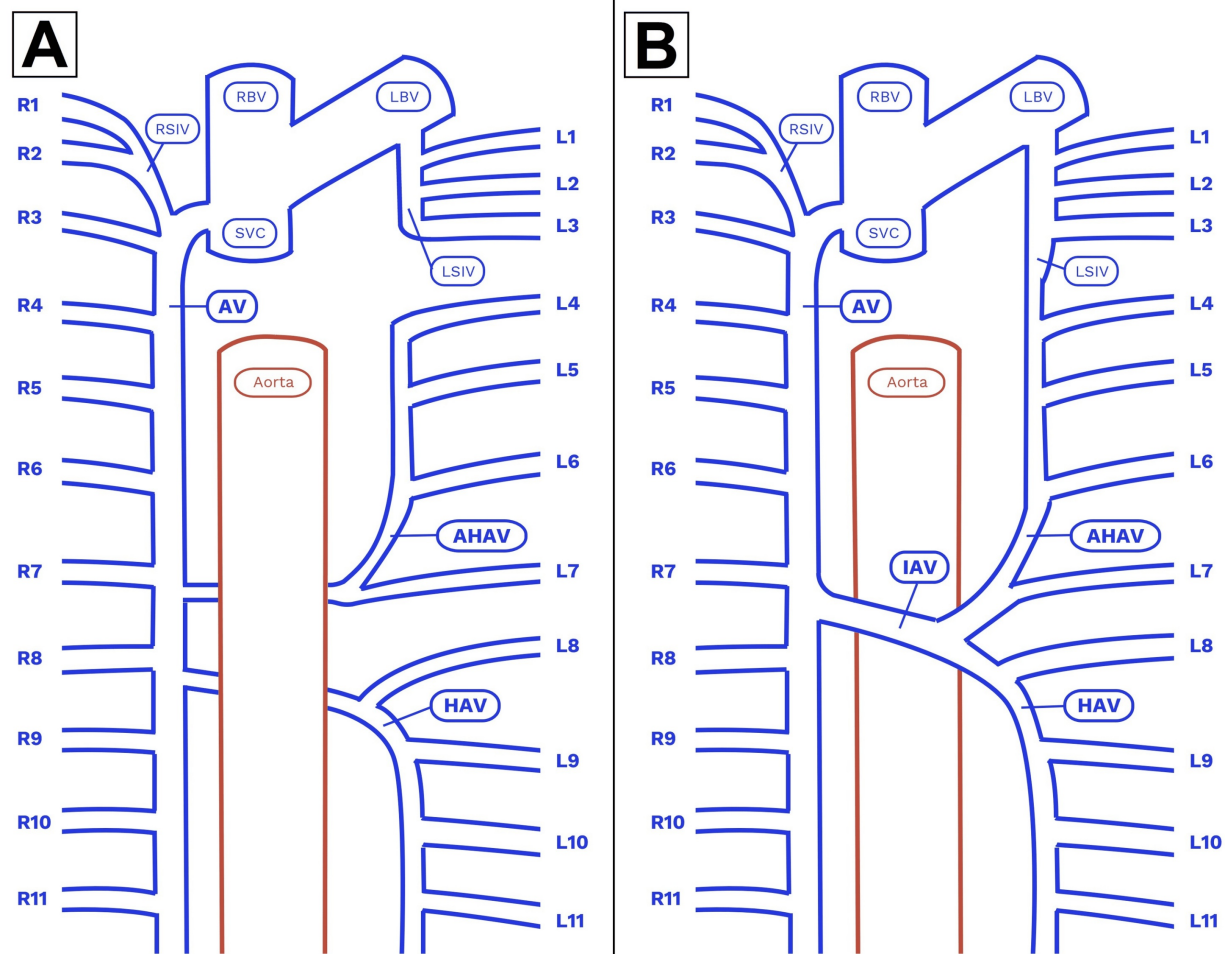
Diagrams of the normal azygos system and the observed anatomic variation. A: Normal retroaortic course of the azygos system. B: Variant preaortic course of the azygos system. AV: azygos vein; IAV: interazygos vein; HAV: hemiazygos vein; AHAV: accessory hemiazygos vein; RSIV: right superior intercostal vein; LSIV: left superior intercostal vein; RBV: right brachiocephalic vein; LBV: left brachiocephalic vein; SVC: superior vena cava. Intercostal veins are labeled numerically. Image Credits: Jay Bauman.

In the course of dissecting the posterior mediastinum region of the thorax, an abnormal structure was observed crossing the ventral surface of the descending thoracic aorta in a transverse orientation (Figure 1). Following the structure to the left side of the posterior thoracic wall revealed that it was continuous with the vertical component of the hemiazygos vein. The hemiazygos vein in this individual received drainage from the left posterior intercostal veins 8-11 (Figure 2).

Next, the course of the accessory hemiazygos vein was examined. It was found to travel vertically on the left posterior chest wall, as expected, where it received drainage from the left intercostal spaces 4-7. However, inferior to the point where it received the left seventh intercostal vein, the accessory hemiazygos vein turned to the right and anastomosed with the transverse venous channel ventral to the aorta. There was also a communication between the accessory hemiazygos vein and the left superior intercostal vein; however, this type of variation is relatively common, with an estimated 57% prevalence rate [2].

Following the preaortic anastomosis to the right side of the thorax confirmed that it communicated with the azygos vein at the level of the intervertebral disc between the eighth and ninth thoracic vertebrae. At this point, the structure was identified as an "interazygos vein" based on a literature search of these anatomical characteristics. Further dissection confirmed that this vessel was the only communication between the left and right sides of the azygos system in this individual; there were no additional retroaortic anastomoses associated with either the hemiazygos or accessory hemiazygos veins. No other abnormalities were observed.

## Discussion

This anatomical variation has been named the interazygos vein, yet its precise definition remains unresolved. Early reports described it as either a preaortic hemiazygos vein or preaortic anastomosis [3,8,9]. The term interazygos vein first appeared in a case report based on clinical imaging in which the authors defined it as the terminal branch of the hemiazygos vein [5]. However, the second instance of the interazygos vein in the literature interpreted the prefix *inter*- to refer to its passage between the aorta and esophagus [6]. Subsequent case reports have identified their findings as "interazygos veins" without definition or clarification.

Given the lack of standardized terminology, a review of preaortic anastomoses of the hemiazygos veins is warranted. Table 1 [4-16] collects 24 cases described as either a "preaortic anastomosis of the azygos system" or an "interazygos vein" over the last century. Notably, 17/24 reports (71%) featured a single preaortic anastomosis of the hemiazygos vein with no additional retroaortic anastomoses from the left posterior intercostal veins. Only 5/24 cases (21%) described a mix of preaortic and retroaortic anastomoses; however, it is possible this was underreported in the studies based on imaging. Additionally, 8/10 of the cases that reported the sex of the donors were found in males; however, this sample is too small to draw any strong conclusions.

**Table 1:** Morphology of prior cases of preaortic hemiazygos veins. All cases (24) of preaortic hemiazygos anastomoses or interazygos veins reported in the last century. Each report was evaluated for sex of the donor (nr = not reported), the total number of anastomoses from the hemiazygos system to the azygos vein, whether each anastomosis was anterior or posterior to the aorta, and the modality by which the case was identified.

Year	Authors	Case #	Sex	Total hemiazygos anastomoses	Preaortic anastomoses	Retroaortic anastomoses	Modality
1934	Seib [4]	1	nr	1	1	0	Dissection
1934	Seib [4]	2	nr	1	1	0	Dissection
1934	Seib [4]	3	nr	1	1	0	Dissection
1934	Seib [4]	4	nr	1	1	0	Dissection
1934	Seib [4]	5	nr	1	1	0	Dissection
1934	Seib [4]	6	nr	1	1	0	Dissection
1934	Seib [4]	7	nr	2	2	0	Dissection
1948	Morton [8]	1	nr	2	1	1	Dissection
1948	Morton [8]	2	nr	1	1	0	Dissection
1952	Butler and Balankura [9]	1	nr	2	1	1	Dissection
1952	Butler and Balankura [9]	2	nr	1	1	0	Dissection
1952	Butler and Balankura [9]	3	nr	1	1	0	Dissection
1983	Smathers et al. [5]	1	M	1	1	0	CT
1996	Celik et al. [6]	1	M	1	1	0	Dissection
1997	Gilkeson et al. [7]	1	F	1	1	0	CT, X-ray
1999	Ozbek et al. [10]	1	M	2	1	1	Dissection
2002	Ozdemir et al. [11]	1	M	7	1	6	Dissection
2004	Das and Paul [12]	1	M	1	1	0	Dissection
2007	Pyrzowski et al. [13]	1	M	1	1	0	Dissection
2009	Nishie et al. [14]	1	F	1	1	0	CT
2016	Ntombela et al. [15]	1	nr	1	1	0	Dissection
2016	Ntombela et al. [15]	2	nr	5	1	4	Dissection
2020	Shiri and Madadi [16]	1	M	2	2	0	Dissection
2025	Present case	1	M	1	1	0	Dissection

Upon review of the case reports, the use of the phrase interazygos vein to describe this anatomic variation should ultimately be re-evaluated. It is not standard anatomic vernacular - the horizontal venous channel that extends from the hemiazygos vein to the azygos vein is typically considered an extension of the hemiazygos [1,2]. Further, the prefix "inter-" is ambiguous in its anatomical relationships; it could refer to either the connection between the azygos and hemiazygos veins or to the position between the aorta and the esophagus. Lastly, this review revealed that the term interazygos vein encompasses a range of morphological variations, mixing preaortic and retroaortic anastomoses. The original terminology of preaortic hemiazygos vein or preaortic anastomosis is a more precise, if wordier, description of this phenomenon.

The azygos venous system develops embryologically from the paired posterior cardinal veins [17]. These veins are also known as the caudal cardinal veins because they drain the caudal or tail portion of the embryo. As the inferior vena cava develops and takes over the role of venous return, the posterior cardinal veins shrink in diameter and move medially. The thoracic portion of the right posterior cardinal vein becomes the azygos vein, and that of the left posterior cardinal vein becomes the hemiazygos and accessory hemiazygos veins. Additionally, transverse anastomoses appear anterior to the vertebral column, all by the 7th week of development. At no point in development are there preaortic anastomoses that later regress, and thus the appearance of this variation has been speculated to be atavistic [4].

Radiologists should be cognizant of this rare venous variation to avoid mistaking it for thoracic aortic aneurysms, mediastinal tumors, or enlarged lymph nodes [7,14]. Further, surgeons should be cautious of the potential for hemorrhage during nearby procedures [6]. Education and awareness of preaortic anastomoses of the hemiazygos veins are therefore vital to ensuring the best possible patient outcomes.

## Conclusions

A preaortic anastomosis of the hemiazygos vein is a rare but clinically relevant anatomical variation. It is sufficiently large to be mistaken for other structures in imaging and is at risk of being damaged during adjacent procedures due to its unexpected placement and thin walls. Raising awareness of this variation among surgeons and radiologists is consequently important to reduce misdiagnoses and surgical complications in the mediastinum. Preaortic anastomoses of the hemiazygos vein have become known as "interazygos veins." While this name is evocative, it is also ambiguous and thus should be reconsidered in favor of a more descriptive alternative.
